# Investigation of *LGALS2* expression in the TCGA database reveals its clinical relevance in breast cancer immunotherapy and drug resistance

**DOI:** 10.1038/s41598-023-44777-1

**Published:** 2023-10-14

**Authors:** Song He, Zhonghao Ji, Qing Zhang, Xiwen Zhang, Jian Chen, Jinping Hu, Ruiqing Wang, Yu Ding

**Affiliations:** 1 Department of Laboratory Animals, College of Animal Sciences, Jilin University, Changchun, Jilin 130062 People’s Republic of China; 2 Department of Basic Medicine, Changzhi Medical College, Changzhi, 046000 Shanxi People’s Republic of China; 3 The Eye Center in the Second Hospital of Jilin University, Ziqiang Street 218#, Nanguan District, Changchun, Jilin 130041 People’s Republic of China

**Keywords:** Cell biology, Breast cancer, Tumour biomarkers, Tumour immunology

## Abstract

Breast cancer (BRCA) is known as the leading cause of death in women worldwide and has a poor prognosis. Traditional therapeutic strategies such as surgical resection, radiotherapy and chemotherapy can cause adverse reactions such as drug resistance. Immunotherapy, a new treatment approach with fewer side effects and stronger universality, can prolong the survival of BRCA patients and even achieve clinical cure. However, due to population heterogeneity and other reasons, there are still certain factors that limit the efficacy of immunotherapy. Therefore, the importance of finding new tumor immune biomarker cannot be emphasized enough. Studies have reported that *LGALS2* was closely related to immunotherapy efficacy, however, it is unclear whether it can act as an immune checkpoint for BRCA immunotherapy. In the current study, changes in *LGALS2* expression were analyzed in public datasets such as TCGA-BRCA. We found that *LGALS2* expression was associated with immune infiltration, drug resistance and other characteristics of BRCA. Moreover, high *LGALS2* expression was closely related to immunotherapy response, and was associated with methylation modifications and clinical resistance for the first time. These findings may help to elucidate the role of LGALS2 in BRCA for the development and clinical application of future immunotherapy strategies against BRCA.

## Introduction

There is no doubt that cancer remains one of the leading causes of death in most countries around the world^1,2^. According to GLOBOCAN 2020 data, there are about 2.3 million new cases of breast cancer (BRCA) in women worldwide, accounting for 11.7% of all malignant tumors^3^. BRCA has become the most prominent cause of mortality in women worldwide, with new cancer cases projected to increase from 24% in 2018 to more than 46% by 2040^4,5^. Triple negative breast cancer (TNBC) is a type of BRCA without the expression of estrogen receptor (ER), progesterone receptor (PR) and human epidermal growth factor receptor 2 (HER2). Compared with other subtypes, TNBC is more prone to recurrence and metastasis, and has a lower survival rate^6,7^. It is worth noting that after receiving conventional treatments such as surgical resection, radiotherapy, and chemotherapy, there are still some patients who fail to benefit from the treatment^8,9^. In addition, the emergence of drug resistance and other phenomena during the treatment process also reduce the efficacy of the clinical treatments. Therefore, it is crucial to find a useful biomarker that can be used for clinical diagnosis/prognosis, with a potential for reducing clinical drug resistance.

Immunotherapy refers to a treatment method that artificially enhances and/or rebuilds the immune system to prevent and resist infection when the patient's body is in a low or hyperactive immune state^10,11^. Studies have demonstrated the therapeutic effects of immunotherapy in BRCA, STAD, LIHC, GBM and other cancer types^7,12–15^. The current immunotherapy regimen for tumors mainly includes monoclonal antibodies, tumor vaccines and non-specific immunotherapy, the purpose of which is to balance the immune system, so that cancer cells are eliminated without inducing autoimmune inflammation^16,17^. In addition, the abnormal expression of CTLA4, TIM-3, PD-1, TIGIT, HVEM and other immune checkpoint molecules is associated with many diseases. Immunotherapy with check point inhibitors increases the aggressiveness of the host's immune system against tumor cells by inhibiting the binding of programmed death receptors and their ligands^18–21^. At present, a large number of immune checkpoints and T cells are in the clinical and preclinical development stages, providing new reference for future tumor treatment strategies^22–24^.

Galectin-2 (LGALS2) is a homodimer consisting of 130 amino acids and a member of the galectin family^25^. They are known to bind to β-galactoside and contain at least one carbohydrate recognition domain (CRD), which plays an important role in many physiological and pathological processes such as cell adhesion, apoptosis, inflammatory response, and tumor metastasis^26–28^. There are currently 11 known human galectins and 15 known animal galectins^29,30^. Galectin-1, -3, -7, -8 and -9 are closely related to tumor immune escape^31^. LGALS2 is also closely related to immunotherapy response and plays an active role in cancer therapy^32–34^.

Based on public databases such as TCGA and METABRI, this study analyzed the expression levels of LGALS2 and the clinicopathological characteristics, diagnosis and prognosis, immune infiltration, etc. of BRCA patients. The results showed that LGALS2 was lowly expressed in BRCA patients (*P* < 0.001), and patients with higher LGALS2 expression had longer survival time (*P* = 0.014), especially LGALS2 had a better diagnostic potential in TNBC patients (AUC = 0.787). Analyses related to immune infiltration, GO and KEGG analysis, GSVA, enrichment analysis, etc. indicated that LGALS2 participated in the immune response of BRCA. Single-cell sequencing further demonstrated that LGALS2 was specifically highly expressed in T cells and could serve as a biomarker for immunotherapy response in BRCA patients.

At the same time, LGALS2 mRNA expression was negatively correlated with LGALS2 promoter methylation level and DNA methyltransferase expression level, and LGALS2 was associated with reduced IC50 values of several clinically used anticancer drugs. In conclusion, LGALS2 and BRCA have potential immunotherapeutic value. Therefore, regulating LGALS2 may be a novel strategy for the treatment of BRCA patients and LGALS2 might be a novel biomarker for BRCA immunotherapy.

## Materials and methods

### BRCA datasets

The Cancer Genome Atlas (TCGA), the Molecular Taxonomy of Breast Cancer International Consortium (METABRIC), the Gene Expression Omnibus (GEO), the Cancer Cell Line Encyclopedia (CCLE), the cBioPortal database and Connectivity Map (CMap) were used to obtain gene expression and pertinent prognostic and clinicopathological data for BRCA patients. In addition, all data were downloaded from public databases and analyzed using the R (version 4.0.3) and R Bioconductor.

### Functional enrichment analysis

In the TCGA dataset (https://portal.gdc.com), we downloaded RNA-sequencing expression (level 3) profiles and clinical information related to BRCA patients. According to the expression level of *LGALS2* gene in the TCGA-BRCA dataset, they were divided into two groups: high (n = 551) and low (n = 550). The R package Limma was used to study the differentially expressed mRNAs. Additionally, Adjusted *P* < 0.05 and Log2 (Fold Change) > 1 or < − 1 was defined as the threshold for the differential expression of mRNAs. We analyzed Gene Ontology (GO) function of the underlying mRNAs and enriched Kyoto Encyclopedia of Genes and Genomes (KEGG) pathway by using the ClusterProfiler package in R^35^.

### Gene set variation analysis (GSVA)

We obtained the immune gene list from the Gene Set Enrichment Analysis (GSEA) (http://www.gsea-msigdb.org/). We calculated each BRCA sample's functional enrichment score using default parameters in R. With the pheatmap package in R, we mapped the enrichment results on a heatmap. Pearson correlation was used to determine the correlation between LGALS2 expression and immune responses.

### Analysis of single‐cell clusters

GSE161529 was obtained from the Gene Expression Omnibus (GEO) database (https://www.ncbi.nlm.nih.gov/geo/) and processed using the Seurat package in R^36^. Genes expressed in more than three cells were considered as expressed, and each cell had to express 200 genes. The FindVariableFeatures function was used to identify the most variable genes from raw UMI counts. The variable genes were used in Principal components analysis (PCA). With a resolution of 0.6, the function FindClusters revealed shared nearest neighbor based on PCA using the first 20 principal components. Two-dimensional representations of the cell states were obtained using Uniform Manifold Approximation and Projection (UMAP) dimensional reduction analysis. Based on the CellMarker database (http://xteam.xbio.top/CellMarker/) and existing literature, the significant genes were used to assign cluster identity to the cell types^37^.

### Correlation analysis of methylation expression

In the TCGA dataset (https://portal.gdc.com), we downloaded RNA-sequencing expression (level 3) profiles and illumina human methylation 450 states related to BRCA. In this study, differentially expressed mRNAs were visualized using the R package ggplot, and the data was transformed to Log2 (Fold Change)^38^.

### Correlation analysis of IC50

In the TCGA dataset (https://portal.gdc.com), we downloaded RNA-sequencing expression (level 3) profiles and clinical information related to BRCA. Using Genomics of Drug Sensitivity in Cancer (GDSC), the chemotherapeutic response was predicted for each sample using the pRRophetic package in R. An estimation of the half-maximal inhibitory concentrations (IC50) was performed by using ridge regression with all parameters set to their default values^39^.

### Small molecule targeted drugs screening of *LGALS2* in BRCA

The Connectivity Map (CMap) (http://www.broad.mit.edu/cmap) was used to search for potential small molecule targeted drugs. Those small molecule drugs with |score|> 0.2 and *P* < 0.05 were recognized as the potential therapeutic drugs targeting *LGALS2* in BRCA^40,41^.

### Statistical analyses

All statistical analyses were performed using the R (version 4.0.3) software. Kaplan–Meier survival curves were constructed and compared with log-rank tests. The Spearman correlation analysis was used to examine correlations among variables without normal distributions. Data were analyzed between two and multiple groups by Student's *t* test and one-way ANOVA. *P* < 0.05 was considered to be statistically significant. (ns, *P* ≥ 0.05, *P < 0.05, **P < 0.01, ***P < 0.001, ****P < 0.0001).

## Results

### LGALS2 expression associates with diagnosis and prognosis in BRCA patients

**Figure 1:**
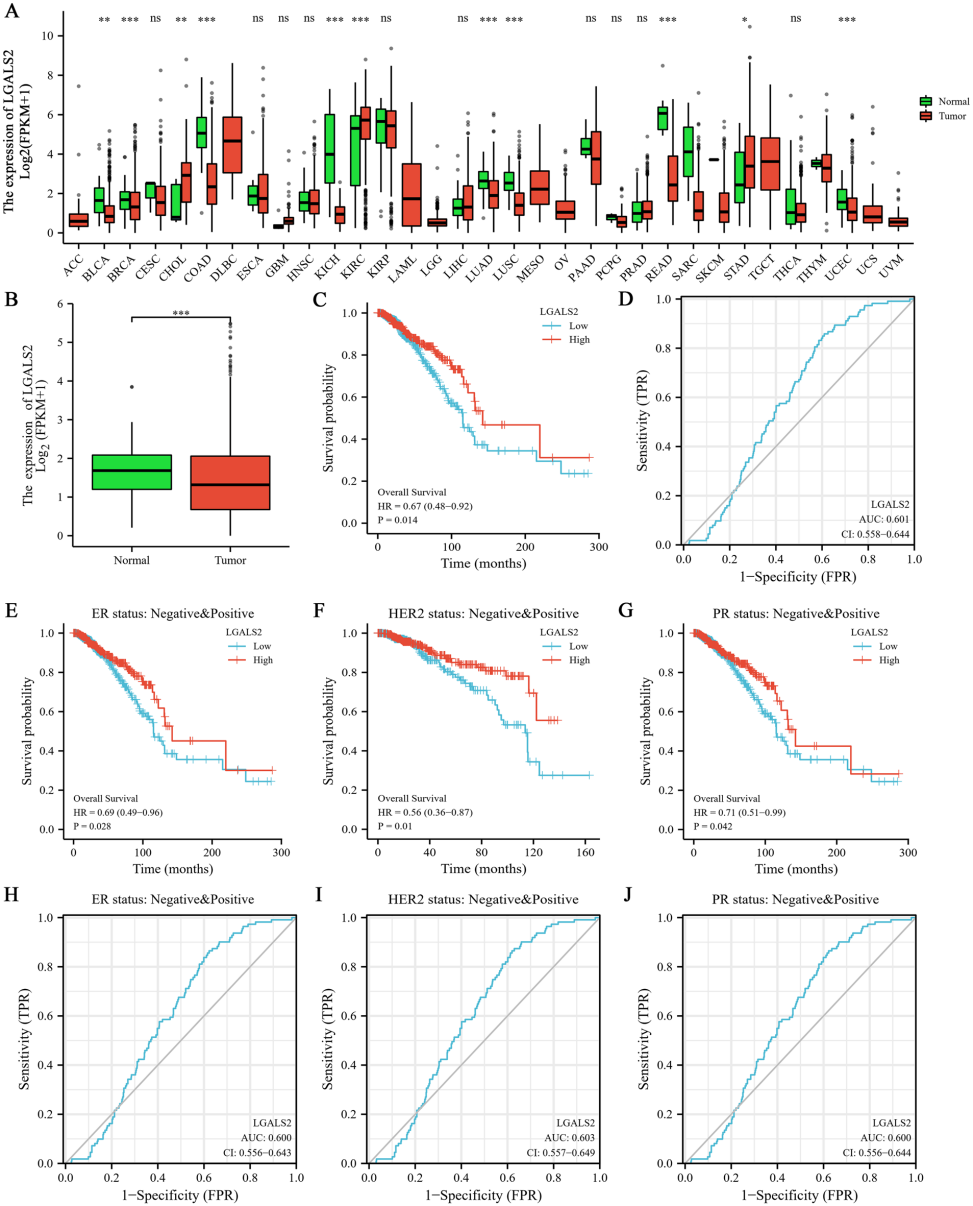
Transcriptional alterations and diagnostic/prognostic value of *LGALS2* in BRCA patients. (**A**) Expression of LGALS2 in paracancers and tumors in TCGA database. The significance of the difference was tested with an unpaired student’s *t* test. (**B**) LGALS2 expression in BRCA patients based on the TCGA database. The significance of the difference was tested with an unpaired student’s *t* test. (**C**) KM analysis of the diagnostic value of LGALS2 in TCGA database. (**D**) ROC analysis of the prognostic value of LGALS2 in TCGA database. (**E**–**G**) KM analysis of overall survival of LGALS2 in patients with different subtypes of BRCA. (**H**–**J**) ROC analysis of prognostic of LGALS2 in patients with different subtypes of BRCA. ns, *P* ≥ 0.05, *P < 0.05, **P < 0.01, ***P < 0.001, ****P < 0.0001.

The TCGA database was used to analyze the expression of *LGALS2* in various normal and tumor tissues. The results showed that *LGALS2* expression was statistically significant among 11 groups including BLCA (*P* < 0.01) (Fig. 1A), and was much lower in tumor groups, especially in BRCA (*P* < 0.001) (Fig. 1B). In addition, the expression levels of *LGALS2* in different tissues (n = 29) and different human breast cancer cell lines (n = 68) were also analyzed based on the CCLE database, which was found to be consistent with the TCGA database results (Fig. S1A, B). Meanwhile, the above cell lines were classified into subtypes and our analyses showed no significant differences between these groups (*P* > 0.05) (Fig. S1C)^42–44^. And then, we further analyzed the diagnostic and prognostic value of *LGALS2* in BRCA. The Kaplan–Meier (KM) curve indicated that BRCA patients with high *LGALS2* expression had better survival rates (*P* = 0.014) (Fig. 1C), also in ER, HER2 and PR patients (*P* < 0.01) (Fig. 1E–G). Moreover, the receiver-operating characteristic (ROC) curve showed the diagnostic value of *LGALS2* in BRCA patients (AUC = 0.601) (Fig. 1D) and their different subtypes (Fig. 1H–J). implying the potential value of *LGALS2* in the diagnosis and prognosis of BRCA.

### Analysis of various clinical factors associated with *LGALS2* expression

**Figure 2:**
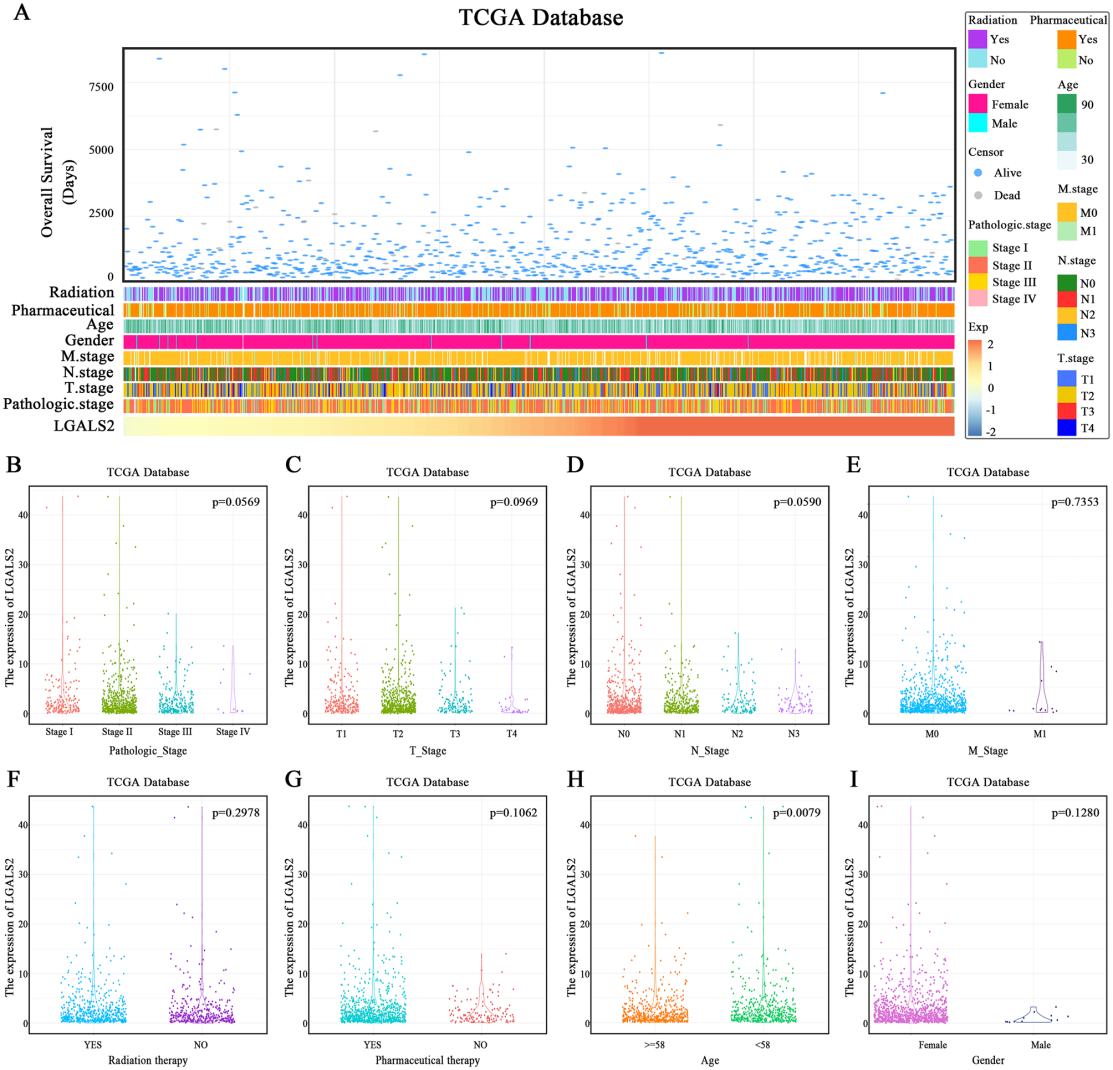
Analysis of various clinical factors associated with *LGALS2* expression. (**A**) The landscape of *LGALS2*-related clinicopathological features of BRCA in TCGA database. (**B**–**I**) *LGALS2* and various clinicopathological features of BRCA in TCGA databases. (**B**–**D**) LGALS2 was not significantly different in Pathologic-, T-, N-stage in TCGA database. The significance of the difference was tested by one‐way ANOVA. (**E**–**I**) LGALS2 was not significantly different in M-stage, radiation therapy, pharmaceutical therapy and gender in TCGA database. The significance of the difference was tested with an unpaired student’s *t* test. (**H**) there was a significant difference between LGALS2 expression and patient age in TCGA databases. The significance of the difference was tested with an unpaired student’s *t* test.

The clinical patients with different expression of *LGALS2* showed the distinct patterns of clinical and pathological characteristics. The change in *LGALS2* expression with chemotherapy or radiotherapy, age and gender, pathologic-stage, T-stage, N-stage, M-stage and survival status are shown in Fig. 2A. The expression of *LGALS2* was not statistically significant in groups with pathological-, T-, N- or M-stage, radiotherapy, drug treatment (Fig. 2B–G) and gender (Fig. 2I), but except for the age group (*P* < 0.01) (Fig. 2H). The clinical factors associated with *LGALS2* expression (e.g., AJCC-stage, age, gender, race, etc.) were also extracted from the cBipPortal database to complement the results of the TCGA database (Fig. S2)^45^. Consistent with the prior results, *LGALS2* expression levels were found to significantly differ only with patient diagnosis age (*P* < 0.05) and race (*P* < 0.001). The results of this study did not reveal a statistically significant difference based on the clinical stage.

### *LGALS2* has diagnostic value for TNBC

**Figure 3:**
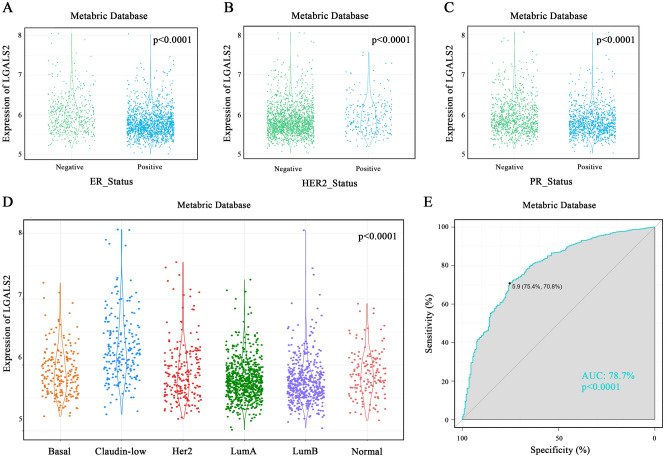
Diagnostic analysis of *LGALS2* expression with TNBC. (**A**–**C**) Expression of *LGALS2* in ER, HER2, PR. LGALS2 was enriched in the negative subtype of BRCA in the Metabric database. The significance of the difference was tested with an unpaired *t* test. (**D**) *LGALS2* was highly expressed in claudin-low subtype of BRCA in the Metabric database. The significance of the difference was tested by one‐way ANOVA. (**E**) The ROC curve showed the high‐expression specificity of *LGALS2* in TNBC in the Metabric database.

Studies have reported that LGALS2 could be used as a therapeutic target for TNBC^33^. However, there are still some data limitations in the TCGA database, thus, the METABRIC database was used to analyze the expression of *LGALS2* in the BRCA subtypes. When molecular markers were detected in various types of BRCA, the expression level of *LGALS2* was significantly elevated in ER-negative, HER2-negative, and PR-negative groups (*P* < 0.0001) (Fig. 3A–C). We then investigated *LGALS2* distribution in different subtypes defined by the METABRIC database. Results showed that *LGALS2* was significantly enriched in the Claudin-low subtype compared to the other subtypes (*P* < 0.0001) (Fig. 3D). *LGALS2* expression specificity was assessed using ROC curves. The area under the curve (AUC) was up to 78.7% in the METABRIC database (*P* < 0.0001) (Fig. 3E). It appeared that *LGALS2* was significantly enriched in TNBC, suggesting its diagnostic potential as a biomarker and providing a reference for the clinical diagnosis of TNBC.

### LGALS2 regulates immune responses in a T cell-dependent manner

**Figure 4:**
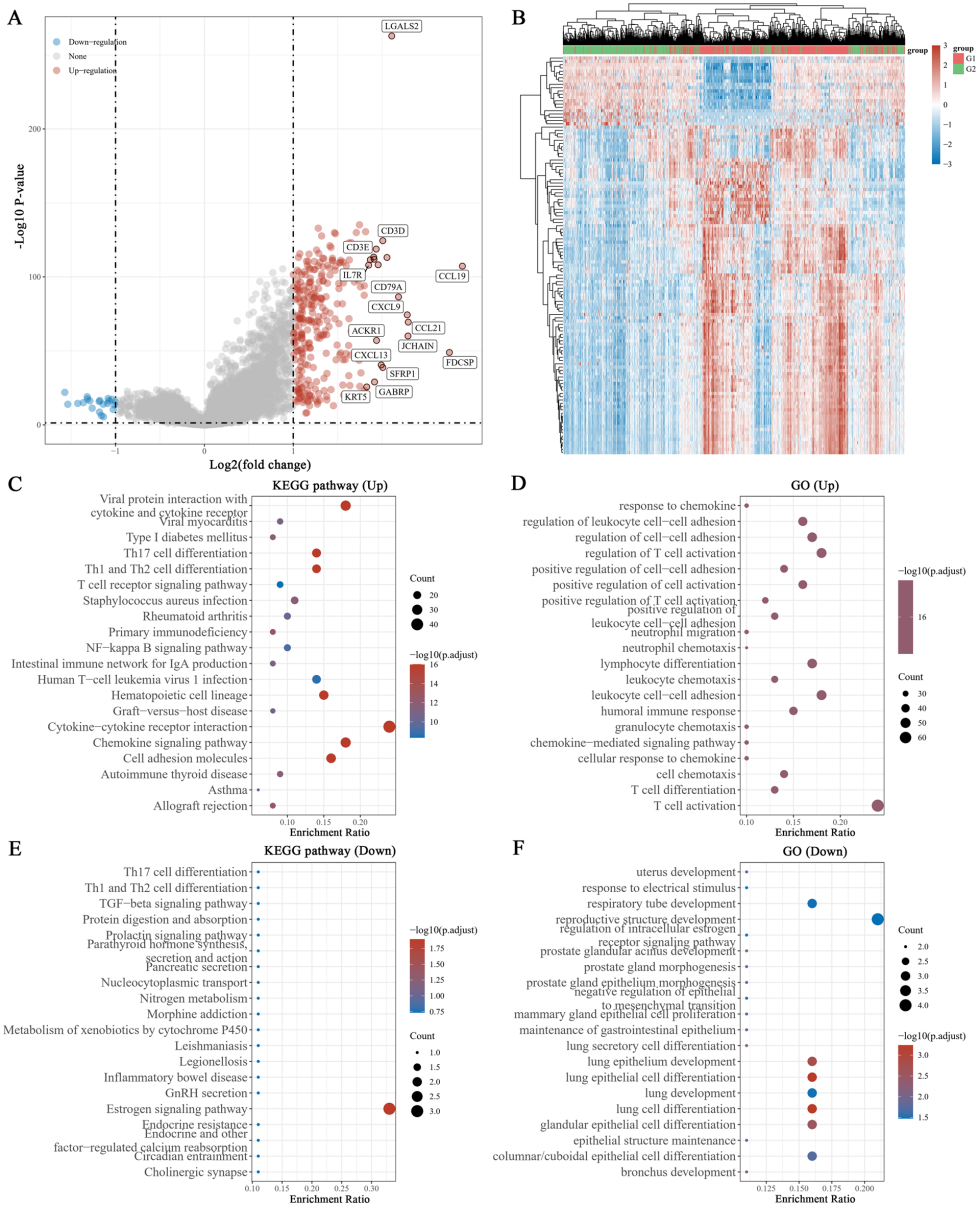
GO and KEGG analysis of *LGALS2*. (**A**) Volcano plot of differentially expressed genes. Red dots indicate upregulated genes, blue dots indicate downregulated genes, grey dots indicate not significant. (**B**) Heat map showing the differentially expressed genes, and the different colors represent the trend of gene expression. The top 50 up-regulated genes and top 50 down-regulated genes were shown in this figure (**C**,**E**) KEGG enrichment analysis. (**D**,**F**) GO enrichment analysis. Colors represent the significance of differential enrichment, the size of the circles represents the number of genes, the larger the circle, the greater the number of genes. (|LogFC|> 1, Adjusted *P* < 0.05).

*LGALS2* expression was investigated in the TCGA database using the LIMMA R package to explore its potential biological function in BRCA. It generated 300 genes, and 279 genes were upregulated, 21 genes were downregulated (|logFC|> 1, adjusted *P* < 0.05) (Fig. 4A,B). In the GO and KEGG analyses, LGALS2 functions were primarily related to cytokine-cytokine receptor interaction, viral protein interaction with cytokine and cytokine receptor, chemokine signaling pathway, T cell activation, leukocyte cell–cell adhesion, regulation of T cell activation, etc. (Fig. 4C–F). Based on these findings, combined with the previous results related to immune infiltration T cells and its association with *LGALS2* expression, we speculated that *LGALS2* might be closely associated with immunotherapy outcome and contribute to immune response in BRCA patients.

### *LGALS2* expression positively correlates with T cell-mediated immune responses

**Figure 5:**
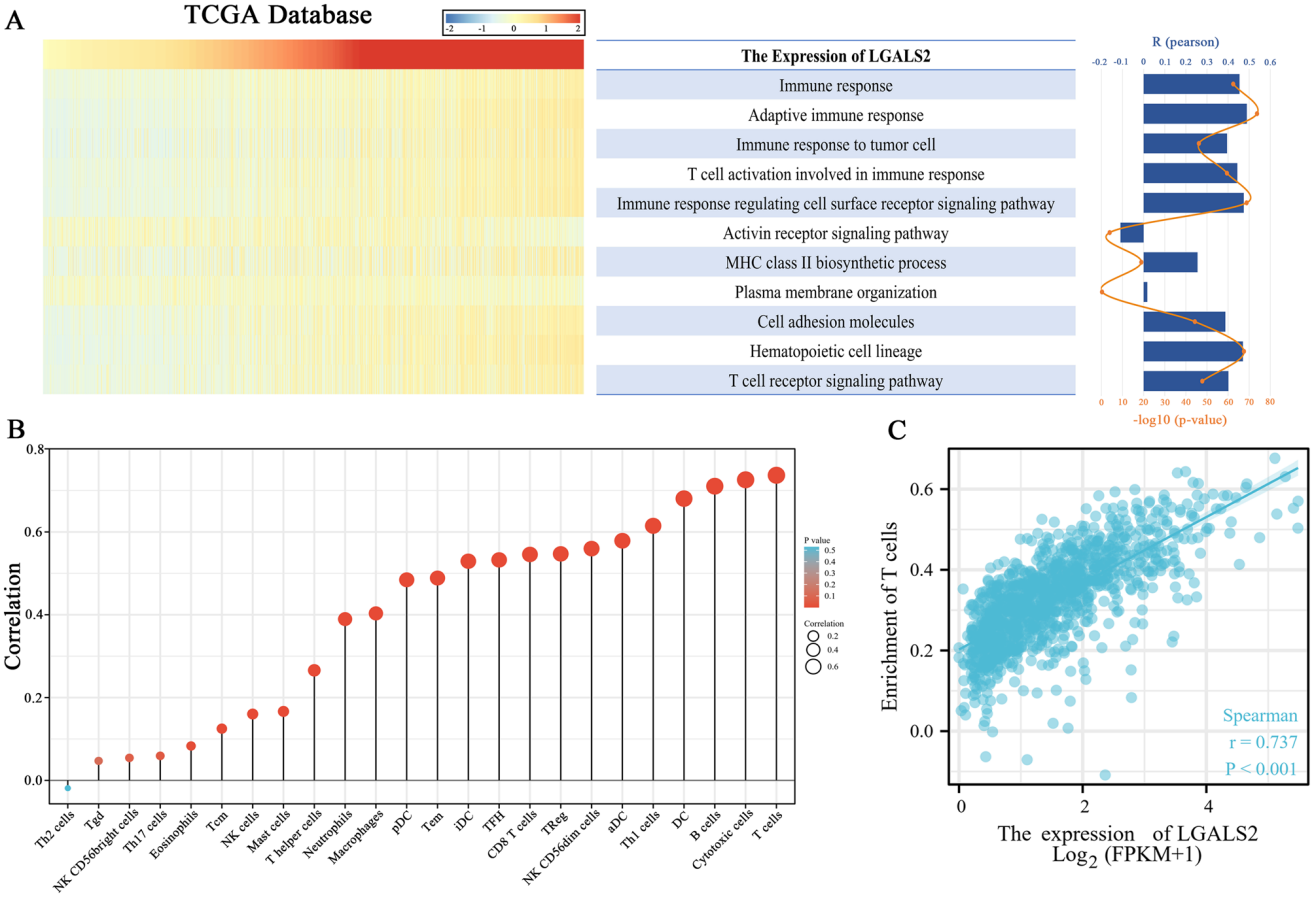
Correlation analysis between *LGALS2* and immune function. (**A**) Heatmap showing LGALS2 expression and the enrichment scores of immune functions of each patient in TCGA, and the column and line graph on the right shows the R and P values for correlation analysis. (**B**) Analysis of the correlation between LGALS2 and immune cells. The size of the circle represents the correlation, with the larger the circle the higher the correlation. (**C**) Scatter plot of LGALS2 and T cell enrichment. LGALS2 expression levels were positively correlated with T cell enrichment. The significance of the difference was tested by Spearman correlation analysis.

Cancer cells die immunologically due to lymphocyte activation (including NK cells, T cells, and B cells) and the release of chemokines and cytokines^46,47^. Therefore, we investigated the effects of *LGALS2* expression on immune pathways and cytokines. TCGA gene set variation analysis (GSVA) was used to determine the immune process enrichment score. Based on correlation analysis between *LGALS2* expression and enrichment score, *LGALS2* expression was positively correlated with most immune functions, but not with activin receptor signaling pathway and plasma membrane organization (Fig. 5A). *LGALS2* expression was associated with various immune cells as shown in Fig. 5B, and was most closely associated with T cells. Moreover, the immune infiltration data showed a higher degree of enrichment of *LGALS2* and T cells (*P* < 0.001, r = 0.737) (Fig. 5C). The above results indicated that *LGALS2* expression was associated with T cell activity and immunotherapy response in BRCA patients.

### *LGALS2* positively correlates with established cancer immune checkpoints

**Figure 6:**
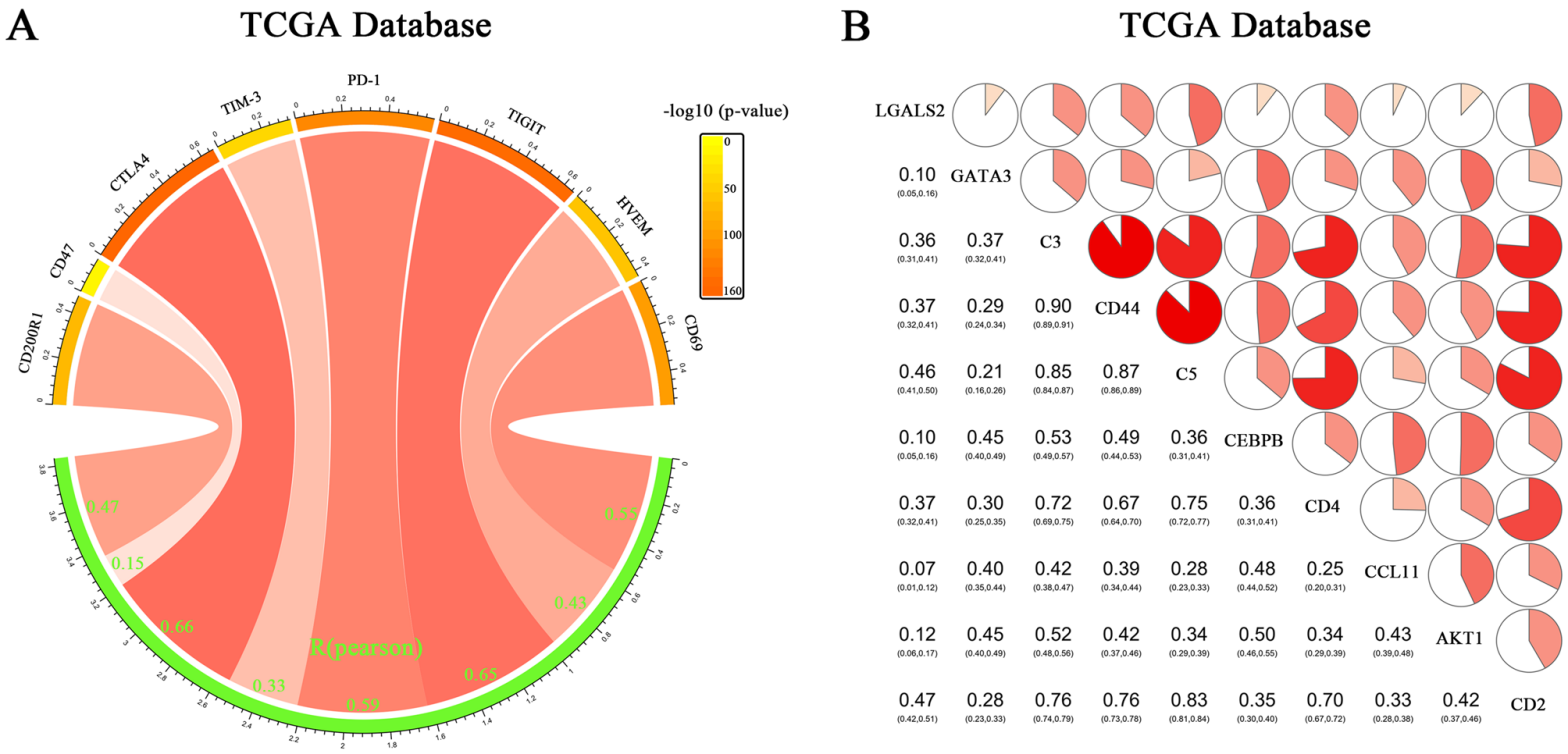
Analysis of the correlation between *LGALS2* and T cell immunity and inflammation. (**A**) Pearson correlation between *LGALS2* and inhibitory immune checkpoints. The color of the band represented the *P*‐value. The correlation was tested by Pearson correlation analysis. (**B**) Correlation matrix of *LGALS2* and inflammatory‐related metagenes. The bottom left showed the correlation coefficient. which are shown as a scale of the pie charts. The correlation was tested by Pearson correlation analysis.

In previous studies, LGALS2 was shown to be involved in tumor immunity^48^, thus we investigated the relationship between *LGALS2* expression in the TCGA dataset and immune checkpoints, including CD200R1, CD47, CTLA4, TIM-3, PD-1, TIGIT, HVEM and CD69. *LGALS2* expression showed a strong relationship with these immune checkpoints^18,19,49^ (Fig. 6A). Additionally, there was a positive correlation between *LGALS2* expression and inflammatory-related metagenes (including GATA3, C3, CD44, C5, CEBPB, CD4, CCL11, AKT1, CD2)^50–54^ (Fig. 6B). Furthermore, these results indicated that *LGALS2* expression was associated with tumor immunotherapy and could serve as a biomarker for response to immunotherapy.

### T cells express high levels of *LGALS2*

**Figure 7:**
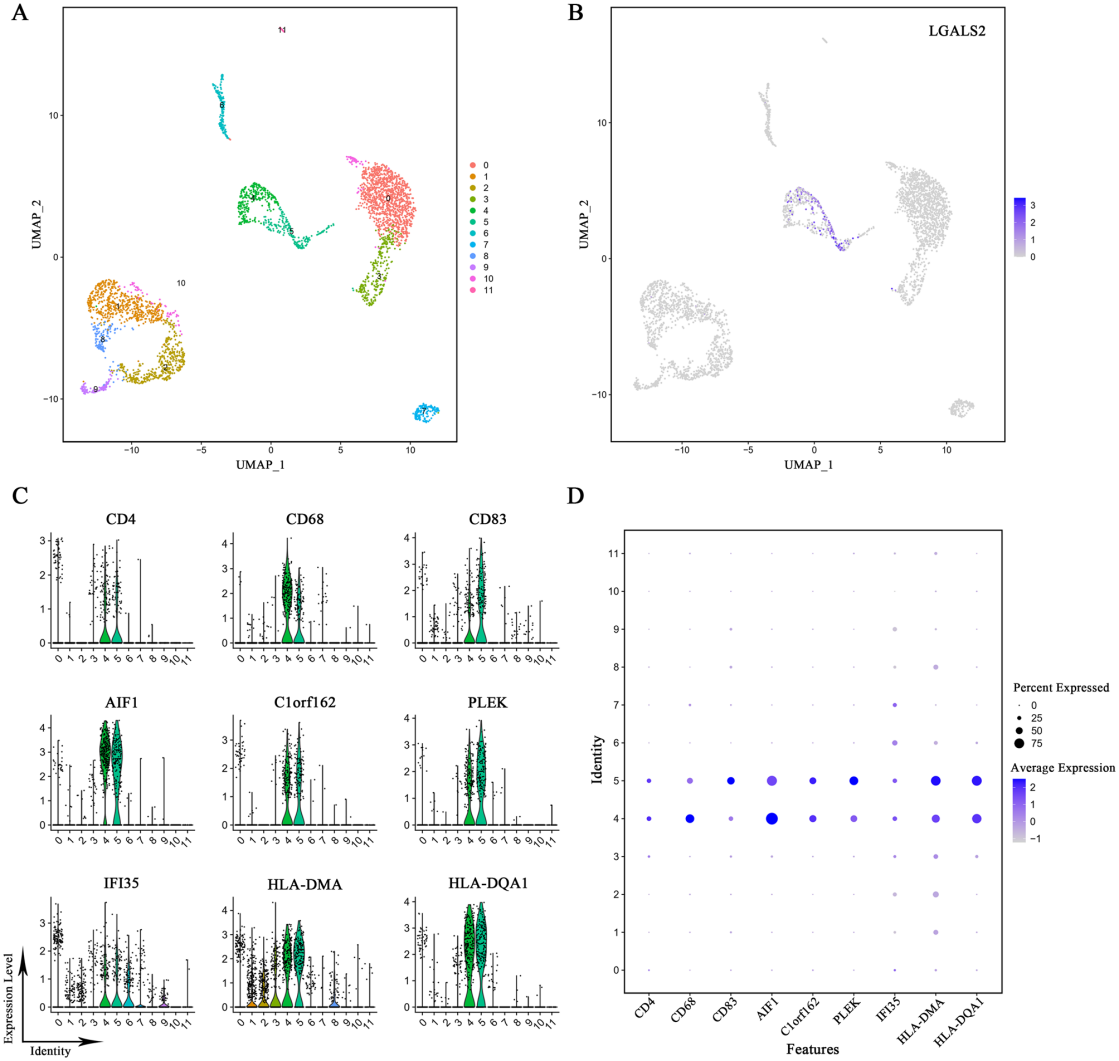
Single-cell sequencing analysis of *LGALS2*. (**A**) Single-cell sequencing analysis showing the cellular subtypes of BRCA, based on the GEO database (GSE161529). (**B**) *LGALS2* is highly expressed in cluster 4 and 5. (**C**,**D**) Expression of T cell markers in different subtypes. Verification of cluster 4 and cluster 5 as T cells.

We analyzed public datasets from the GEO database using R for single-cell sequencing. UMAP dimensionality reduction analysis was used to obtain a two-dimensional representation of cell state and 12 clusters were created (Fig. 7A). We found that LGALS2 was mainly enriched and highly expressed in the clusters 4 and 5 (Fig. 7B). Cellular markers enabled us to identify the cluster 4 and cluster 5. Based on the expression of multiple cellular markers, such as CD4, CD68, CD83 and so on^55–57^, clusters 4 and 5 were categorized as T cells (Fig. 7C,D).

In the subsequent analyses, we showed that *LGALS2* expression was closely associated with the immune system and interacted with T cells. Thus, upregulating *LGALS2* expression was associated with the activation and differentiation of T cells, induction of immune responses and promotion of the necrosis or apoptosis of breast cancer cells.

### *LGALS2* expression level positively correlates with methylation modification

**Figure 8:**
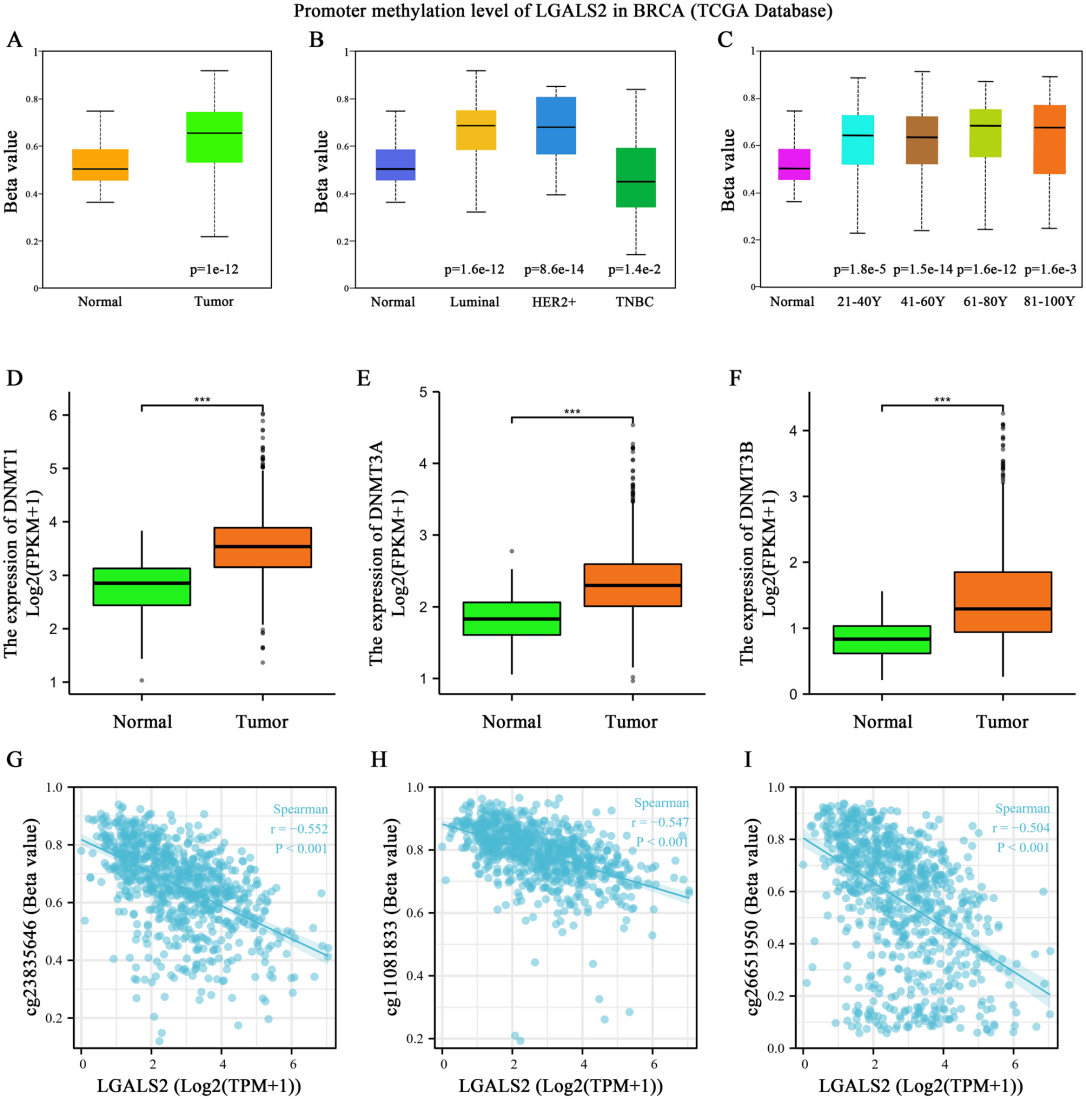
Methylation-related analysis of *LGALS2*. (**A**) Promoter methylation level of *LGALS2* in BRCA. The significance of the difference was tested with an unpaired student’s *t* test. (**B**) *LGALS2* promoter methylation expression based on BRCA subclasses. The significance of the difference was tested with an unpaired student’s *t* test. (**C**) *LGALS2* promoter methylation expression based on age of BRCA patients. The significance of the difference was tested with an unpaired student’s *t* test. (**D**–**F**) Relationship between DNA methyltransferases (DNMT1, DNMT3A and DNMT3B) and *LGALS2* expression in BRCA patients. The significance of the difference was tested with an unpaired student’s *t* test. (**G**–**I**) Methylation probe linked to *LGALS2* in BRCA patients. The significance of the difference was tested by Spearman correlation analysis. ns, *P* ≥ 0.05, *P < 0.05, **P < 0.01, ***P < 0.001, ****P < 0.0001.

In light of the importance of DNA methylation in regulating gene expression^58,59^, we examined *LGALS2*-associated methylation level. A significant increase in promoter methylation of *LGALS2* was observed in tumor samples as compared to the normal group (*P* < 0.0001) (Fig. 8A), and this was also significantly associated with the tumor type and patient age (Fig. 8B,C), which were inversely correlated with *LGALS2* mRNA expression level (Fig. 1B). At the same time, the expression levels of DNA methyltransferases (DNMT) (including DNMT1, DNMT3A and DNMT3B) in the normal and tumor samples from TCGA database were also determined. The results showed that DNMT was highly expressed in breast cancer (*P* < 0.0001) (Fig. 8D–F). Furthermore, the results suggested that methylation occurred 2400–2700 bp downstream of *LGALS2* transcription start sites (cg23835646, cg11081833 and cg26651950) (*P* < 0.001) (Fig. 8G–I). Due to this, DNMT might affect the transcription process by adding methyl groups to *LGALS2* without affecting its sequence, thereby reducing the expression of *LGALS2*, and the methylation modification of *LGALS2* might be one of the reasons for its decreased mRNA expression.

### *LGALS2* expression associates with clinical sensitivity to anticancer drugs

**Figure 9:**
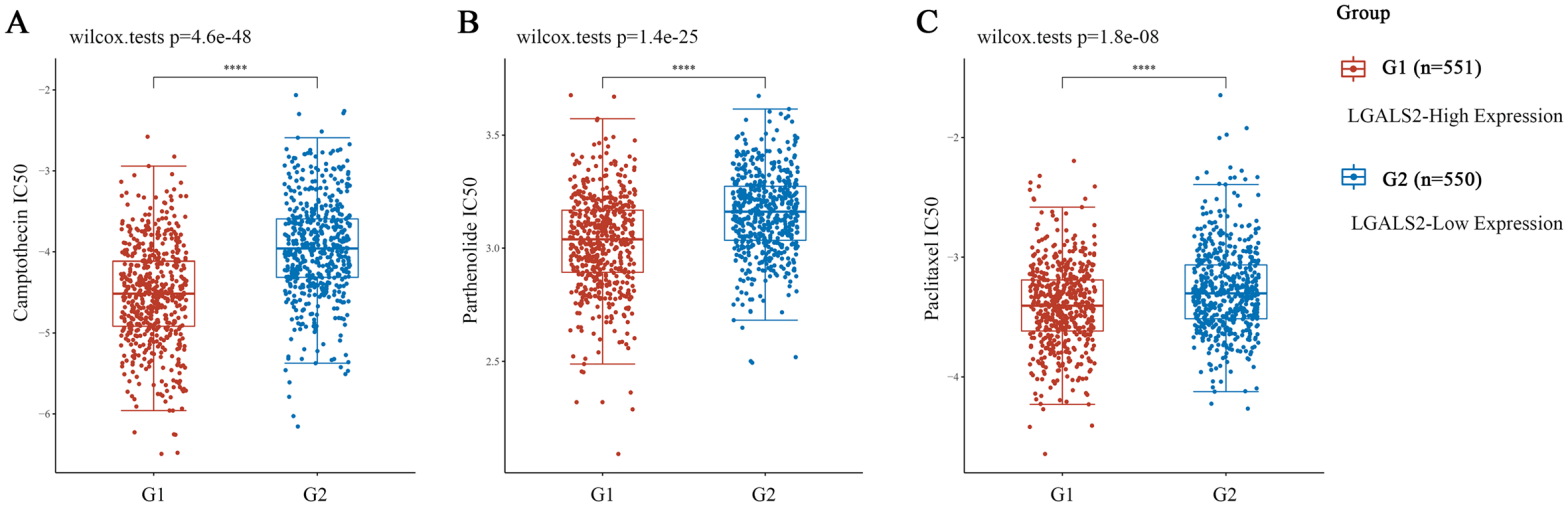
Evaluation of *LGALS2* and IC50 for clinical drugs. (**A**–**C**) LGALS2 reduces IC50 of clinical drugs and reduces drug resistance. The significance of the difference was tested with an unpaired student’s *t* test. ns, *P* ≥ 0.05, *P < 0.05, **P < 0.01, ***P < 0.001, ****P < 0.0001.

To further explore LGALS2 as a potential therapeutic target in BRCA, we performed CMap analysis and obtained 15 compounds with positive and negative correlations, respectively (Fig. S4A)^60,61^. It has been reported that cancer patients often suffer from drug resistance, which leads to relapses and reduced survival rates^62^. The role of *LGALS2* expression in breast cancer drug resistance was therefore studied. Surprisingly, we found a negative correlation between *LGALS2* expression and resistance in BRCA (*P* < 0.0001) (Fig. S3). High levels of *LGALS2* were associated with a significant reduction in the IC50 values of several clinical anticancer drugs (including camptothecin, parthenolide and paclitaxel)^63–71^ (Fig. 9). Meanwhile, CMap analysis further evaluated to validate the above drugs. The results also showed that these drugs were significantly associated with *LGALS2* expression in BRCA (*P* < 0.01) (Fig. S4B–E). Therefore, *LGALS2* expressed levels might be associated with the increased sensitivity of cancer cells to clinical drugs and longer survival of cancer patients.

## Discussion

Breast cancer is the number most common cause of cancer related mortality in women globally, and TNBC accounts for about 10% to 15% of that^72^. Although the patient's condition is improved after surgical resection, radiotherapy, chemotherapy and other therapeutic interventions, adverse reactions such as metastasis and drug resistance contribute to the overall unsatisfactory treatment outcomes^73,74^. Compared with other therapeutic strategies, cancer immunotherapy causes fewer side effects and is more universal, which prolongs the survival time of patients and even achieves clinical cure, and thus is a promising treatment strategy^75,76^. However, there are still some limitations such as tumor immune escape, tumor mutation burden, and adaptive immune resistance, which suppress the therapeutic potential of cancer immunotherapy^38,77^. Therefore, it is crucial to find a new biomarker for evaluating immunotherapy response in breast cancer treatment.

In this study, we found that *LGALS2* expression was low in breast cancer patients and was lower in the breast tissue relative to other tissues, and patients with high *LGALS2* expression had a better prognosis (*P* = 0.014). Furthermore, we show that *LGALS2* could be used as a diagnostic marker for breast cancer (AUC = 0.601), and has a better diagnostic value in TNBC (*P* < 0.0001). The ROC analysis showed that the AUC-value was 0.787. The above findings suggest that *LGALS2* has a good prognostic value for BRCA patients, especially for TNBC patients.

However, in the analysis of *LGALS2* and related clinicopathological factors based on the TCGA and cBioPortal database, there was no statistically significant correlation between *LGALS2* expression and the commonly used clinical stages. Braud VM et al.^78^ reported that KLRB1 was a predictive marker for the survival status of patients, but was not related to the degree of tumor cell invasion, and our findings were consistent with them. In addition, the current common tumor staging is based on the length of the tumor, the status of the ipsilateral axillary lymph node, and the presence of distant metastasis^79,80^. Therefore, we speculated that *LGALS2* might not be directly involved in the migration and development of breast cancer. Of course, data is an important factor affecting the results, including the data collection process, the size and quality of the data, the regional distribution of patients, and the heterogeneity and bias of the data^81,82^.

DNA methylation is often dysregulated during tumorigenesis and cancer progression, which are manifested by the reduction of the overall methylation level of the genome and the inactivation of the local DNA regions^83,84^. Whether the low expression of LGALS2 in breast cancer patients was related to DNA methylation modification has not yet been reported. Here, we found that *LGALS2* promoter methylation levels were high in the tumor groups (*P* < 0.001) and were closely related to the tumor subtypes and patient age (*P* < 0.05). At the same time, DNA methyltransferases (DNMT) (including DNMT1, DNMT3A, DNMT3B) were also highly expressed in tumors (*P* < 0.001). Namely, LGALS2 expression was negatively correlated with *LGALS2* promoter methylation and DNA methyltransferase. As a result, we speculated that DNMT regulated methylation modifications in the *LGALS2* promoter region to inhibit the transcription of *LGALS2*, resulting in decreased LGALS2 expression. Moreover, the results suggested that methylation occurred 2400-2700 bp downstream of *LGALS2* transcription start sites (cg23835646[TSS + 2561], cg11081833[TSS + 2683], cg26651950[TSS + 2445]) (*P* < 0.001). Therefore, reducing the methylation level of the *LGALS2* promoter, thereby reversing the occurrence and development of breast cancer, could serve as a new strategy for breast cancer treatment. However, further research is warranted to verify the above findings.

Previous studies reported that LGALS2 induced T lymphocyte apoptosis, improved colitis, and prevented preeclampsia^27,85^. During cancer immunotherapy, T cells play a key role in the antitumor response and are closely associated with the effective inhibition of immune checkpoints^86,87^. In this study, GO and KEGG analysis, GSVA, enrichment score, etc. revealed that LGALS2 was associated with increased T cell infiltration (*P* < 0.001) and had a positive regulatory relationship with immune response. The data used in these analyses were all obtained from the average of multiple data samples, but we could not obtain information regarding inherent tumor heterogeneity. However, single-cell sequencing solves this problem well, with higher sensitivity and accuracy, and more reliable results. Cell markers such as CD4, CD68, AIF1, PLEK, and others were used to identify T cell clusters^56,88^, and we found *LGALS2* to be specifically highly expressed in T cell clusters. The above findings suggested that *LGALS2* regulated T cells to induce immune response in the body and participated in tumor immunotherapy.

The combination of immunotherapy and chemotherapy (so-called chemo-immunotherapy) has demonstrated excellent therapeutic effects in the clinical treatment of cancer patients^89,90^, but adverse reactions such as drug resistance significantly limit the therapeutic efficacy of the combination strategy^91,92^. The main cause of cancer recurrence is the development of multidrug resistance, which in turn is associated with increased expression of efflux transporters, epithelial-to-mesenchymal transformation and breast cancer stem cell resistance^93^. In addition, it has been demonstrated that the formation of an immunosuppressive niche increased the occurrence of drug resistance and suppressed the antitumor effects of T cells, reducing the efficacy of immunotherapy^94^. We therefore examined the role of LGALS2 in clinical drug resistance based on the GDSC database. The results showed that high *LGALS2* expression was associated with a lower IC50 value of 3 clinical antitumor drugs, including camptothecin, which enhanced their drug sensitivity and reduced the occurrence of clinical drug resistance. Subsequently we also used CMap to analyse the correlation between these 3 drugs and *LGALS2* in BRCA, and gratifyingly this result also showed that these drugs were significantly correlated with *LGALS2* expression. Of course, the results of the database have to be verified by further wet experiments.

Twyman-Saint Victor et al.^95^ reported that the up-regulation of PD-L1 in melanoma cells was an important factor in the development of drug resistance and was related to T cell exhaustion. Interestingly, therapy- and resistance-related changes in T cells might constitute biomarkers of tumor response. Moreover, immune checkpoints such as PD-1/PD-L1, TIM-3, and TIGIT are known to play important roles in the treatment of NSCLC, OV, SKCM, BRCA, and have been recognized as biomarkers for immunotherapy response^96,97^. Through enrichment analysis and immune score, Jiang et al.^98^ identified that the expression level of STC2 was significantly positively correlated with the infiltration level of B cells, T cells and other immune cells in various cancer cells, and was significantly correlated with the sensitivity towards certain drugs, and suggested STC2 as a promising target for tumor immunotherapy. Therefore, *LGALS2* could also serve as a potential marker for breast cancer immunotherapy.

Our study systematically and comprehensively validates that *LGALS2* can be used as a diagnostic/prognostic marker for breast cancer, especially a good diagnostic marker for TNBC, and that it participates in tumor immunotherapy by regulating T cells. The result that high *LGALS2* expression was associated with reduced occurrence of clinical tumor therapy resistance was even more intriguing for us. However, our study suffered from some limitations. First of all, this study solely relied on existing public data, thus necessitating further experimental evidence to verify and clarify the molecular mechanism of LGALS2 in BRCA. Secondly, the low expression of *LGALS2* in BRCA was related to the methylation modification of its promoter region and the ability of *LGALS2* to reduce the occurrence of clinical drug resistance was also identified for the first time in this study. However, further experimental and clinical studies are needed to confirm the above findings. Lastly, although the results of this study supported the involvement of *LGALS2* in tumor immune regulation and immunotherapy, the underlying molecular mechanism needs further investigation.

## Conclusion

In summary, our study shows that *LGALS2* could be used as a diagnostic and prognostic marker for breast cancer, and that it regulates the biological activity of T cells to participate in tumor immunotherapy and reduces the occurrence of clinical drug resistance in patients. As a novel molecular biomarker for breast cancer treatment, *LGALS2* may enable the development of novel immunotherapy strategies that are of a high clinical relevance in the future. The findings from this study need further validation through in vitro and in vivo experiments to confirm the functions of *LGALS2* and unravel the underlying molecular mechanisms in breast cancer.

### Supplementary Information

Supplementary Figures.

## Data Availability

The datasets analyzed for this study can be found in the TCGA-BRCA project (http://www.cancer.gov/tcga), METABRIC (https://www.mercuriolab.umassmed.edu/metabric), GEO (https://www.ncbi.nlm.nih.gov/geo/query/acc.cgi?acc=GSE161529) and GDSC (https://www.cancerrxgene.org/), CCLE (https://sites.broadinstitute.org/ccle/), cBioPortal (https://www.cbioportal.org/), CMap (https://clue.io/).

## Abbreviations

AUC: Area under the curve
AJCC: American joint committee on cancer
BRCA: Breast cancer
CMap: Connectivity map
CCLE: Cancer cell line encyclopedia
CRD: Carbohydrate recognition domain
ER: Estrogen receptor
GDSC: Genomics drug sensitivity in cancer
GEO: Gene expression omnibus
GO: Gene ontology
GSEA: Gene set enrichment analysis
GSVA: Gene set variation analysis
HER2: Human epidermal growth factor receptor 2
IC50: Half-maximal inhibitory concentration
KEGG: Kyoto encyclopedia of genes and genomes
KM: Kaplan–Meier
METABRIC: Molecular taxonomy of breast cancer international consortium
PR: Progesterone receptor
ROC: Receiver‐operating characteristic
TCGA: The cancer genome atlas
THPA: The human protein atlas
TNBC: Triple negative breast cancer
