# Supplementary material for: Investigation of LGALS2 expression in the TCGA database reveals its clinical relevance in breast cancer immunotherapy and drug resistance
Source: Sci Rep. 2023 Oct 14;13:17445. doi: 10.1038/s41598-023-44777-1 (PMC10576795; doi:10.1038/s41598-023-44777-1)
Supplement: Supplementary file 1 — Supplementary Figures. [file 41598_2023_44777_MOESM1_ESM.docx]

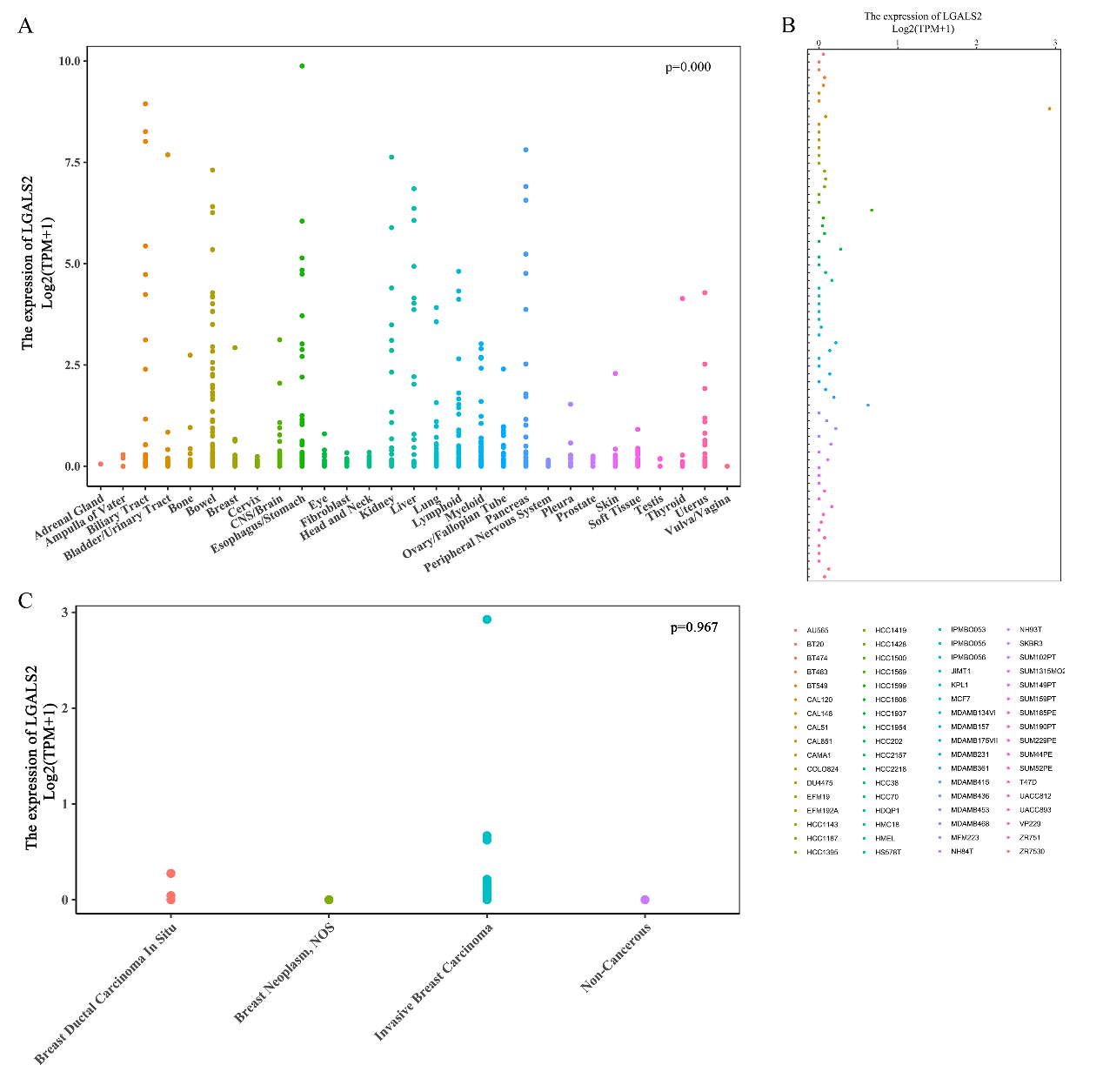


**Figure S1. Expression of *LGALS2* in the CCLE database.** (A) The expression level of LGALS2 was significantly different in 29 tissues (*P*<0.001) and low in breast tissues. The significance of the difference was tested by one‐way ANOVA. (B) Expression of LGALS2 in 68 cell lines in breast tissue. (C) There were no significant differences in LGALS2 among the four different breast cancer cell line subtypes. The significance of the difference was tested by one‐way ANOVA.


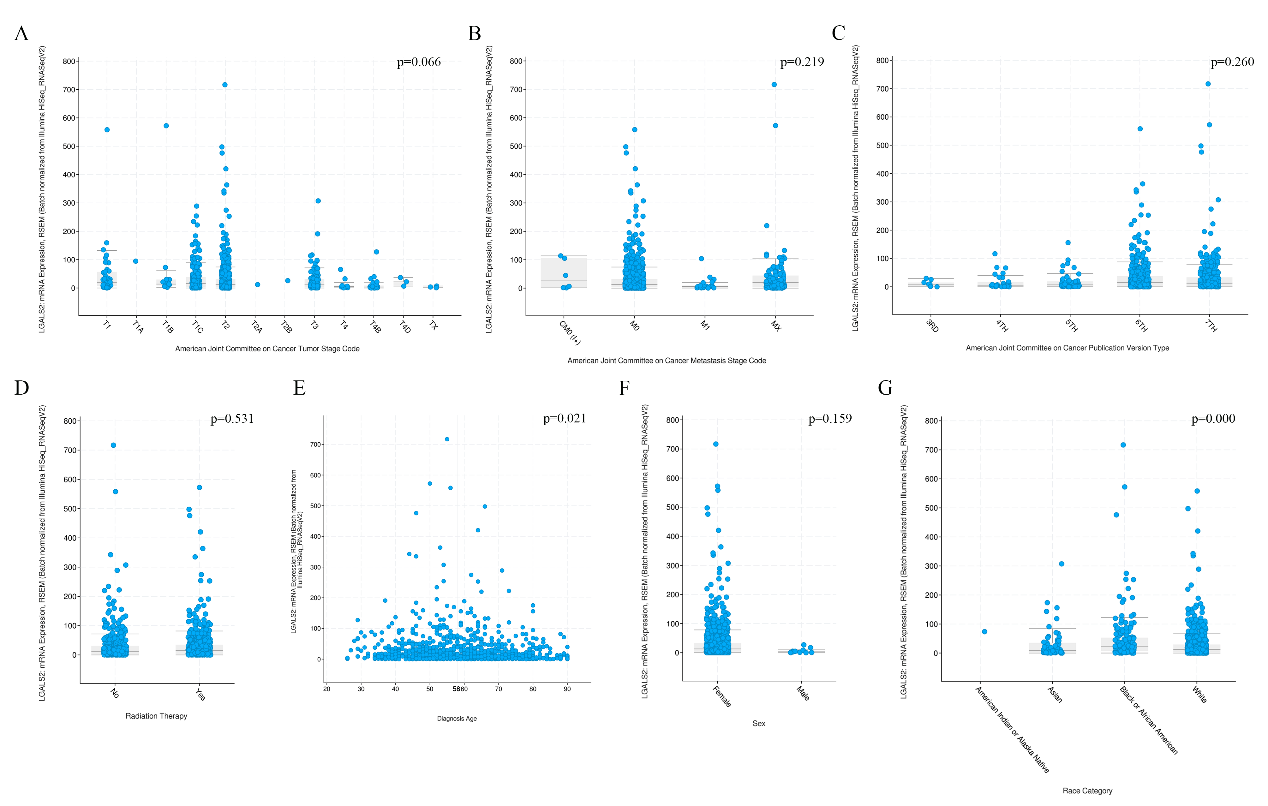


**Figure S2. Analysis of *LGALS2* expression in clinical factors of BRCA based on the** **cBioPortal database.** (A-C) There was no significant difference between LGALS2 in AJCC-tumor stage, AJCC-metastasis stage and AJCC-publication version type. The significance of the difference was tested by one‐way ANOVA. (D, F) There was no significant difference in LGALS2 expression in the presence or absence of radiation therapy, and gender did not affect LGALS2 expression. The significance of the difference was tested with an unpaired student’s *t* test. (E) There was a significant difference between LGALS2 expression levels and patient age (Subgrouped by 58). The significance of the difference was tested with an unpaired student’s *t* test. (G) There was a significant difference between LGALS2 expression levels and patient race. The significance of the difference was tested by one‐way ANOVA.


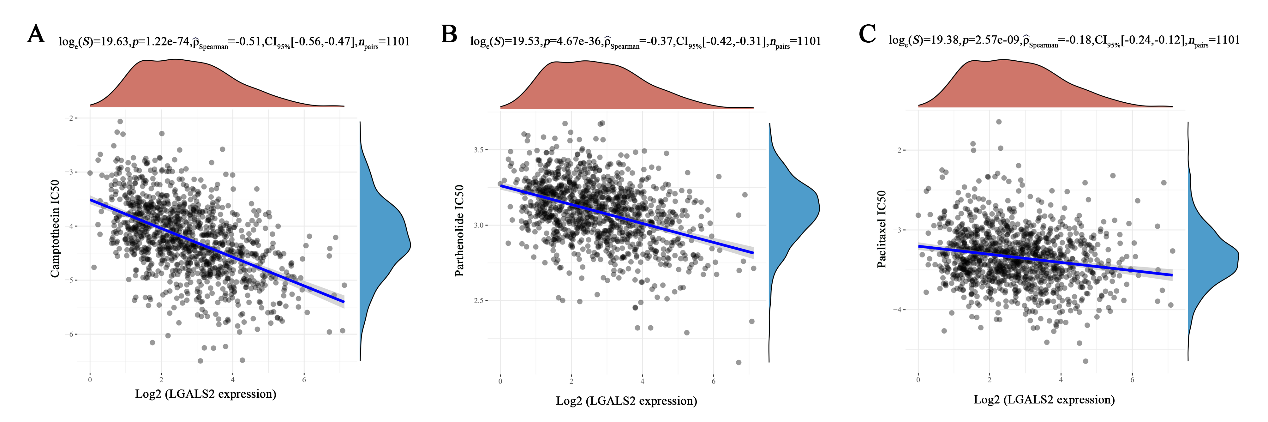


**Figure S3. Assessing the relationship between *LGALS2* and clinical medication. (A-C)** The *LGALS2* expression was negatively correlated with IC50 values of clinical chemotherapeutic agents.


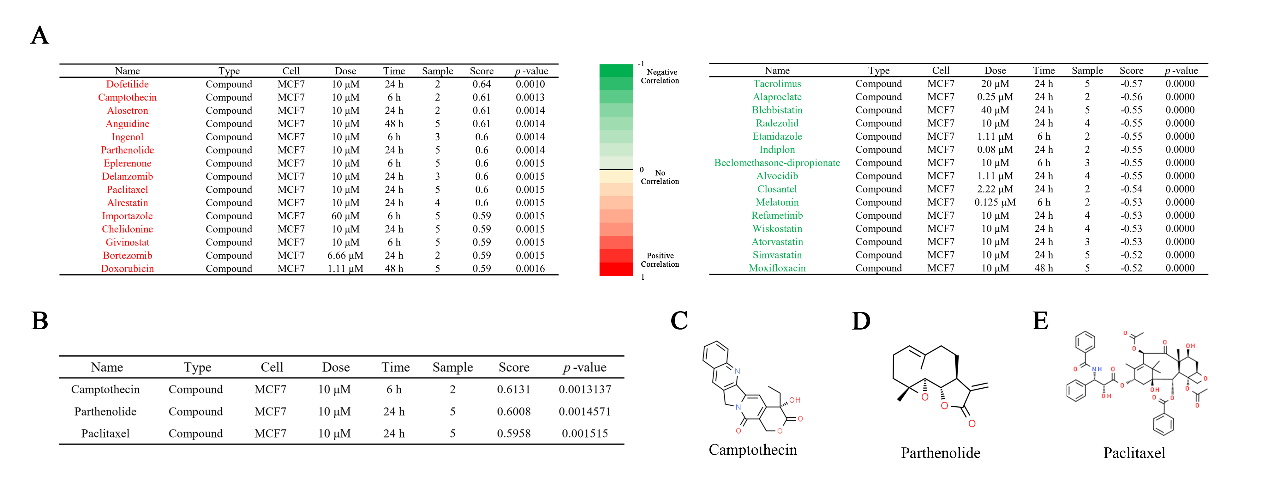


**Figure S4.** **Connectivity map analysis of *LGALS2* compound in BRCA. (A)** CMap analyses for the top and bottom 15 compounds, representing positive and negative correlations for BRCA patients, respectively. The larger the score, the stronger the correlation. **(B)** CMap analysis of 3 compounds with *LGALS2* in BRCA. **(C-E)** The chemical molecular structure of 3 compounds.
